# The Chemistry of Breadmaking

**Published:** 1889-02

**Authors:** 


### THE CHEMISTRY OF BREAD-MAKING.

The transformation of the flour, obtained by grinding wheat, into a soft, spongy, nutritious mass known as bread, although a matter of every day occurrence, is dependent upon some very peculiar and important chemical changes. If flour is mixed with water to form a dough, and dried at the temperature of the air, an unpleasant-tasting mass is obtained, which contains the starch of the flour in an unaltered and insoluble state, and very difficult of digestion ; and to obtain a nutritious and palatable bread it is necessary to induce certain chemical and physical changes in the flour.

Wheat flour is principally composed of starch, and contains, on an average, about sixty-three per cent. of that substance, with fifteen per cent of water. The remainder is made up of small amounts of sugar, fat, casein, and a mixture of various gummy bodies, generally known as gluten. This gluten is an important part of the flour, giving it consistency and flavor, and also rendering it more nutritious. By kneading flour under a gentle stream of water, the starch can be washed away and the gluten separated.

The action of the heat in baking causes certain changes to take place in the starch, by which it is rendered soluble, and to some extent converted into another substance resembling gum, and known as dextrine. The outside of the loaf is altered to a greater extent, forming the crust. These changes have the effect of rendering the bread both nutritious and palatable ; but to prevent its becoming a heavy, solid mass of dried dough, it must be "raised," or inflated with gas, so as to convert it into a light, spongy substance which can be easily masticated and digested.

The gas used for this purpose is always carbonic dioxide, and the best method to develop it in the mass of dough is to set up a vinous or alcoholic fermentation by the addition of yeast. This substance is a most remarkable living organism, which, when introduced into the dough, begins to feed upon the starch, which it changes into alcohol and carbonic acid gas. Owing to the tenacious nature of the dough, the gas cannot escape, but, as it expands, renders it spongy and light. The heat of the baking-oven still further expands the gas, and completes the process, at the same time killing the yeast, and preventing further fermentation. If the fermentation continues too long, it passes over into the acetic variety,the alcohol is changed to vinegar, and the bread "sours."

The alcohol produced in the process is nearly all dissipated in the baking ; but it is an appreciable quantity, and some years ago a company was formed in England to introduce appliances for condensing and saving it. The method was found impracticable, but it created considerable excitement, and one baker advertised to sell his bread "with all the gin in it."

When baking-powder is used as a leavening agent, carbonic-acid gas is formed in the dough as with yeast, only it is set free by the reaction of certain chemicals upon each other, forming salts which remain in the bread, and none of the constituents of the flour are decomposed. A process has also been tried of kneading the dough in closed vessels with water containing carbonic-acid gas in solution under pressure, like soda water ; the supposition being, that when the pressure was removed, the gas would expand, and cause the dough to rise. This aerated bread, as it was called, has been made both in this country and England ; but the method has not been successful, and it is not now in use.

It is interesting to note that the style of oven and method of bread-baking in use in Europe at present has undergone little or no change since the earliest times. The old-fashioned brick oven is still used, and the fire built inside until the necessary heat is obtained, when it is raked out, and the loaves put in its place. Although the method is a clumsy and wasteful one, it must be confessed that the bread produced by the French and German, and especially the Viennese bakers, is unexceptionable in quality. Probably the care used in mixing and kneading has as much to do with the result as the baking itself.

Bread is not often adulterated, but the deficiencies of a poor flour are sometimes covered up by the addition of alum or a minute quantity of sulphate of copper (blue vitrol). These substances render the bread whiter and of a better consistency, but are not only a fraud upon the purse of the consumer, but detrimental to his health as well. Most of the cheaper varieties of baking-powder contain alum, and their use cannot be recommended.

The art of bread-making has been known from the earliest times, and as early as the period of the Jewish exodus the process of leavening was in general use. In the museum at Naples there are exhibited loaves of bread taken from a shop at Pompeii and stamped with the name of the baker. They are perfect in shape, but are quite black, probably from the heat of the volcanic ashes which were cast upon the city from Vesuvius. When properly made, bread is a most wholesome and nutritious food, and, with the other cereal product, forms the principal means of sustenance of a large majority of the human race.

